# Concerning Dental Colleges

**Published:** 1882-01

**Authors:** J. Foster Flagg

**Affiliations:** Philadelphia, Pa.


### ARTICLE V.

### *Concerning Dental Colleges.*

BY J. FOSTER FLAGG, D. D. S., PHILADELPHIA, PA.

This caption, having become somewhat familiar through the efforts of the *Dental Register* (July number,) and of Prof. Joseph Richardson, of the Indiana Dental College, it seems to me appropriate that the discussion of the subject should be continued under it. I recognize that peculiar circumspection should attach to the utterance of views in relation to this matter by anyone who may be thought to be biased by faculty membership, and I therefore desire that my faculty associates shall in no wise be credited with any responsibility for that which I shall offer, but that my conclusions shall be received as purely individual.

I have, for the past few years, noticed the directed tendency of the American Dental Association in its proposed *management* of dental education with feelings of increasing distrust and disgust. I have distrusted it from the appearance of favoritism with which it seemed to me to be possessed, when I was a looker-on and had no special interest in any school, and never expected again to have. I was disgusted with evident effort at usurpation on the part of a committee, the practical knowledge of which, in educational matters, I knew to amount to nothing. I distrusted the truth of the assertions regarding the benefits to dentistry, which were to accrue from the leadings of gentlemen who never had traveled one step in the path they would direct. I was disgusted at the mere mention of coercion, by penal enactments, of those who had conscientious difference of opinion upon an evidently debatable question. I spoke as an individual having no interest, except that which I regarded as the good of my profession, in antagonism to the *spirit* and *tendency* of the movement. I spoke against the *time* requirements as weighed in the scale against *merit*. I spoke in favor of increasing demands for knowledge as the facilities for importation increased. I spoke for that which I regarded as the standard for the gradual elevation of dentistry, as evinced by the growing meaning of its "D. D. S.;" and then I advocated the bestowal of the degree *solely* on the basis of possession by the applicant of the requisite amount of knowledge and the requisite degree of skill.

Of this, it seemed to me, the faculties of the various schools were naturally the best judges. I was not a member of any faculty, but I had been, and had learned what I knew of this work in a very expensive and laborious manner. I knew that the would be dictators had no such work to refer to. I know that the faculties of medical schools had their varied requirements, and that they were regarded as worthy of trust in examinations; I knew of their reputed short-comings; I knew of their endeavors to improve. I knew that the faculties of dental schools had their varied requirements, and I did not feel that the record showed that they were any less trustworthy than their medical brethren; I knew of their shortcomings; I knew of their earnest desires and of their endeavors to improve.

I felt that the good work was going on; that each year showed an advance; that year by year the means for instruction aggregated, and the facilities for the requirement of knowledge, theoretic and practical, were greater; and I

also saw that this was due to the energy and enterprise of those connected with the colleges, and to their full perception of the "needs" for the future welfare of their profession. I saw that this was done while the discussions of the American Dental Association relative to it were practically ignored; I saw that its progress was a thing insured, and that the voice of the committee passed unheeded by the collegiate ear.

The faculty did what they thought best, regardless alike of the meddling or the fulminations of the association. At last came the committee report, in which it was, as has been appropriately said, "*unblushingly*" asserted "that this association claims the right to exercise a general supervision over the whole subject of dental education," which report was adopted, and it was

"*Resolved*, That in order to secure representation in this association dental colleges must, subsequent to October 1st, 1881, require all students entering therein to take two full courses of lectures previous to coming forward for examination and graduation, and must also state these conditions in their next annual announcement."

This extraordinary proceeding has produced results *no less extraordinary*. It has demonstrated that "secured seats" are *not* at a premium. It has demonstrated that representation in the *great supervisory* is not desired by such of the "*reputable*" colleges as regard it disreputable and derogatory to their dignity as educational institutions to barter their birth-right for any such mess of pottage. It has demonstrated that neither the American Dental Association nor its educational committee is, in the least degree, essential to the welfare of a large proportion of the colleges of dentistry. It has produced an evident contempt for the action of both on the part of a large number of the best informed and most advanced members of the profession—men who hold in their hands far more than does the association—the "future" of dentistry; men who, as I have seen it and now see it, have demonstrated a far livelier interest in education than ever has been done by the members of the educational committee; men whose record is of longer standing than is the existence of the association; men who recognize that they largely helped to make it, and who would still have an interest in its legitimate work, but who also recognize that it has had *nothing*, practically, to do with their advancement, and that it has now assumed an attitude which renders it, in their opinion, *an obstruction to their usefulness.—Dent. Cosmos.*

---

